# Prophylactic incisional wound irrigation with aqueous chlorhexidine gluconate vs aqueous povidone iodine for prevention of surgical site infection in elective abdominal surgery

**DOI:** 10.1016/j.infpip.2026.100535

**Published:** 2026-03-30

**Authors:** Nathan Bontekoning, Hannah Groenen, Gerjon Hannink, Markus W. Hollmann, Stijn W. de Jonge, Niels Wolfhagen, Marja A. Boermeester, EPO2CH Study Group, Quirine JJ. Boldingh, Wouter J. Bom, Linda M. Posthuma, Jochem CG. Scheijmans, Bart MF. van der Leeuw, Joost AB. van der Hoeven, Jens-Peter Hering, Dirk JA. Sonneveld, Otto E. van Geffen, Eduard R. Hendriks, Ewoud B. Kluyver, Ahmet Demirkiran, Luc RCW. van Lonkhuijzen, Marcel GW. Dijkgraaf

**Affiliations:** p Amsterdam UMC Location University of Amsterdam, Department of Surgery, Meibergdreef 9, Amsterdam, The Netherlands; q Amsterdam Gastroenterology Endocrinology & Metabolism, Amsterdam, The Netherlands; e Department of Anesthesiology, Albert Schweitzer Hospital, Dordrecht, The Netherlands; f Department of Surgery, Albert Schweitzer Hospital, Dordrecht, The Netherlands; g Department of Anesthesiology, Dijklander Ziekenhuis, Hoorn, The Netherlands; h Department of Surgery, Dijklander Ziekenhuis, Hoorn, The Netherlands; i Department of Anesthesiology, Tergooi MC, Hilversum, The Netherlands; j Department of Surgery, Tergooi MC, Hilversum, The Netherlands; k Department of Anesthesiology, Rode Kruis Hospital, Beverwijk, The Netherlands; l Department of Surgery, Rode Kruis Hospital, Beverwijk, The Netherlands; m Department of Gynecologic Oncology, Amsterdam UMC, University of Amsterdam, Centre for Gynecologic Oncology Amsterdam, Amsterdam, The Netherlands; n Amsterdam UMC Location University of Amsterdam, Epidemiology and Data Science, Amsterdam, The Netherlands; o Amsterdam Public Health, Methodology, Amsterdam, The Netherlands; a Amsterdam UMC Location University of Amsterdam, Department of Surgery, Meibergdreef 9, Amsterdam, The Netherlands; b Amsterdam Gastroenterology Endocrinology & Metabolism, Amsterdam, The Netherlands; c Department of Medical Imaging, Radboud University Medical Center, Nijmegen, The Netherlands; d Amsterdam UMC Location University of Amsterdam, Department of Anesthesiology, Meibergdreef 9, Amsterdam, The Netherlands

**Keywords:** Surgical site infection, Wound irrigation, Povidone iodine, Chlorhexidine gluconate

## Abstract

**Background:**

Incisional prophylactic intra-operative wound irrigation (pIOWI) with an aqueous antiseptic solution effectively reduces surgical site infections (SSIs); however, data on the use of aqueous chlorhexidine gluconate remain limited, and no clinical comparisons with aqueous povidone iodine exist. We therefore compare the efficacy of aqueous chlorhexidine gluconate with aqueous povidone iodine for incisional pIOWI in the prevention SSI in elective abdominal surgery.

**Methods:**

A post hoc analysis of data from the randomised controlled Enhanced PeriOperative Care and Health (EPO_2_CH) trial was conducted, including 699 patients, to assess the effect of aqueous chlorhexidine gluconate compared with aqueous povidone iodine for incisional pIOWI on the incidence of SSI. Multiple imputation with chained equations was used to impute missing values. The association between the type of irrigation and SSI was assessed using inverse probability of treatment-weighted logistic regression.

**Findings:**

Weighted regression analysis showed a statistically non-significant difference in the SSI rate (-3.03%, 95% confidence interval [CI]: -7.04 to 0.98) for pIOWI with aqueous chlorhexidine gluconate (4.05%) vs aqueous povidone iodine (7.08%).

**Interpretation:**

While the SSI rate for pIOWI with aqueous chlorhexidine gluconate showed a lower estimated SSI incidence than with aqueous povidone iodine, the difference was not statistically significant, with wide CIs, indicating uncertainty. Further research is needed to assess the hypothesis that pIOWI with chlorhexidine gluconate, than in povidone iodine, may reduce SSI.

## Introduction

Surgical site infection (SSI) is the most common postoperative complication, significantly increasing morbidity, mortality, and healthcare costs [1,2]. Incisional prophylactic intra-operative wound irrigation (pIOWI) is used to prevent SSI [3]. Our group recently published a systematic review and network meta-analysis, which found high-certainty evidence that incisional pIOWI with an aqueous antiseptic solution is effective for reducing SSI risk compared with no irrigation [4].

Interestingly, most studies have focused on aqueous povidone iodine solutions, while exploring the potential benefits of aqueous chlorhexidine gluconate solutions for pIOWI could be valuable. Chlorhexidine gluconate in alcohol, particularly a higher concentration (2–2.5% chlorhexidine gluconate), has been shown to be more effective than povidone iodine in alcohol as a skin antiseptic for SSI prevention, suggesting that it may also offer advantages in pIOWI [5]. However, current international guidelines on the prevention of SSI do not mention the use of aqueous chlorhexidine gluconate for this purpose since clinical data are limited [[6], [7], [8], [9], [10]]. The guidelines from the US Centers for Disease Control and Prevention (CDC) [7] and the World Health Organization [8,9] specifically recommend aqueous povidone iodine for pIOWI.

Notably, our network meta-analysis identified only one randomised controlled trial (RCT) that investigated an aqueous chlorhexidine gluconate solution for pIOWI [4]. This RCT used a very low concentration of 0.05% chlorhexidine gluconate solution in sterile water and compared its efficacy with an antibiotic solution [11]. The efficacy of higher concentrations of chlorhexidine gluconate remains unclear, and moreover, a direct comparison with povidone iodine in a clinical setting is lacking thus far.

The Enhanced PeriOperative Care and Health (EPO_2_CH) trial evaluated a set of interventions for the prevention of SSI in elective abdominal surgery, collectively known as the EPO_2_CH bundle [[12], [13], [14]]. The bundle did not significantly reduce SSI risk and did not have sufficient statistical power to investigate the effect of individual interventions. Nonetheless, it provides valuable data on the use of aqueous antiseptics (either chlorhexidine gluconate or povidone iodine) for incisional pIOWI.

The aim of this study was to compare the efficacy of aqueous chlorhexidine gluconate with aqueous povidone iodine for incisional pIOWI in the prevention of SSI in patients undergoing elective abdominal surgery by conducting a post hoc analysis of the EPO_2_CH trial using a weighted linear probability model.

## Methods

This study adheres to the Strengthening the Reporting of Observational Studies in Epidemiology (STROBE) guidelines (Supplementary Table A1) [15]. Prospectively gathered data from the EPO_2_CH trial were analysed to compare the efficacy of aqueous chlorhexidine with aqueous iodine for incisional pIOWI for the prevention of SSI.

### Study design and population

The EPO_2_CH trial was an open-label, pragmatic, randomised controlled, parallel-group multi-centre trial. Patients 18 years of age or older were eligible if they were scheduled for elective abdominal surgery with an incision larger than 5 cm. Patients were randomly assigned to the EPO_2_CH bundle or standard care in a 1:1 ratio. The EPO_2_CH bundle consisted of five interventions to prevent SSI: 1) intra-operative high fraction of inspired oxygen (FiO_2_ 0.80); 2) intra-operative goal-directed fluid therapy (GDFT); 3) normothermia (active pre-, intra-, and postoperative warming); 4) peri-operative glucose control and treatment if glucose >10 mmol per litre; and 5) incisional wound irrigation with an aqueous antiseptic agent before wound closure (after closure of the fascia). The choice of agent for pIOWI (0.35%–10% aqueous povidone iodine solution or 1% aqueous chlorhexidine gluconate) was left to the discretion of the surgeon. Standard care was defined based on the discretion of the treating physicians. The primary outcome was SSI incidence within 30 days according to the Centers for Disease Control and Prevention (CDC) definition [16]. The study protocol and main results of the EPO_2_CH trial were previously published [[12], [13], [14]].

### Study cohort

Patients enrolled in the EPO_2_CH trial were allocated to either standard care without pIOWI or a bundle of care including pIOWI. As a result, any comparison between pIOWI and no pIOWI would be heavily confounded by the other interventions in the bundle. Therefore, we were limited to the data of the intervention arm, where all patients were allocated to the bundle and thus received comparable treatment but may have been treated with different pIOWI agents. For the present study, 699 patients from the intervention group of the EPO_2_CH trial who received pIOWI with aqueous chlorhexidine gluconate or aqueous povidone iodine were included.

### Outcomes

The primary outcome of this study was superficial or deep SSI within 30 days after surgery, as defined by the CDC [16]. Secondary outcomes included the separate analysis of superficial SSI and deep SSI. As the intervention concerns incisional irrigation with an aqueous antiseptic agent after fascia closure, the intervention is not expected to affect organ-space infections such as intra-abdominal abscesses or anastomotic leaks, and these were excluded from this analysis.

### Data analysis

There was a low percentage of missing data. There were eight variables with missing values, ranging from 0.1% to 11.9% (Table I). Missing data were assumed to be missing at random, and multiple imputation was performed with chained equations to generate 10 imputed datasets [17,18]. A complete-case sensitivity analysis was performed.

**Table I:** Preweighting patient and procedure characteristics

Characteristic	All patients (*n* = 699)	Povidone iodine (*n* = 445)	Chlorhexidine gluconate (*n* = 254)
Age (years), mean (SD)	63.0 (13.0)	63.8 (12.8)	61.7 (13.3)
Male sex, n (%)	308 (44.1)	168 (37.8)	140 (55.1)
BMI (kg/m^2^), mean (SD)	26.6 (4.7)	26.1 (4.4)	27.5 (5.1)
Current smoker, n (%)	267 (38.5)	183 (41.5)	84 (33.3)
*Missing*	*6*	*4*	*2*
ASA physical status classification, n (%)			
I - II	519 (74.8)	359 (81.4)	160 (63.2)
III - IV	175 (25.2)	82 (18.6)	93 (36.8)
*Missing*	*5*	*4*	*1*
Type of hospital, n (%)			
Academic hospital	361 (51.6)	164 (36.9)	197 (77.6)
Non-academic hospital	338 (48.4)	281 (63.1)	57 (22.4)
Contamination level, n (%)^a^			
I, clean	53 (7.6)	12 (2.7)	41 (16.1)
II, clean-contaminated	612 (87.6)	417 (93.7)	195 (76.8)
III – IV, contaminated and dirty	34 (4.9)	16 (3.6)	18 (7.1)
Procedure duration, hours, mean (SD)	3.3 (1.6)	3.0 (1.4)	3.8 (1.8)
*Missing*	*2*	*2*	*0*
Estimated blood loss, ml, mean (SD)	391 (684)	299 (500)	561 (912)
*Missing*	*83*	*45*	*38*
Surgery indication, n (%)			
Benign	183 (26.6)	66 (14.8)	117 (46.1)
Malignancy	516 (73.8)	379 (84.2)	137 (53.9)
Diabetes, n (%)	111 (15.9)	60 (13.5)	51 (20.1)
Chronic obstructive pulmonary Disease, n (%)	50 (7.2)	28 (6.3)	22 (8.7)
Procedure category, n (%)			
Colorectal surgery	369 (52.8)	272 (61.1)	97 (38.2)
General surgery	107 (15.3)	44 (9.9)	63 (24.8)
Gynaecology	106 (15.2)	100 (22.5)	6 (2.4)
Hepato-pancreato-biliary surgery	99 (14.2)	15 (3.4)	84 (33.1)
Upper gastrointestinal surgery	18 (2.6)	14 (3.1)	4 (1.6)
Goal-directed fluid therapy, n (%)^b^	571 (87.7)	373 (88.8)	198 (85.7)
*Missing*	*48*	*25*	*23*
Hyperoxygenation, n (%)^b^	557 (86.1)	351 (88.4)	213 (82.4)
*Missing*	*52*	*48*	*4*
Normoglycemia, n (%)^b^	311 (44.6)	205 (46.2)	106 (41.7)
*Missing*	*1*	*1*	*0*
Normothermia, n (%)^b^	446 (66.2)	278 (65.4)	168 (67.5)
*Missing*	*25*	*20*	*5*

Patient and procedure characteristics and missing data among the two treatment groups from the original cohort.

SD, standard deviation; BMI, body mass index; ASA, American Society of Anesthesiologists.

a According to the Center for Disease Control wound classification.

b Additional interventions from the EPO_2_CH bundle.

Descriptive statistics were calculated, with means and standard deviations (SDs) reported for continuous variables and numbers and percentages for categorical variables.

Inverse probability of treatment weighting (IPTW) was applied to adjust for potential confounding between the groups treated with aqueous povidone iodine solution and aqueous chlorhexidine gluconate for pIOWI [19]. Propensity scores (PSs) were defined as the probability of receiving pIOWI with aqueous chlorhexidine gluconate, dependent on selected covariates. These PSs were calculated within each imputed dataset separately by using logistic regression, with pIOWI type as the dependent variable and selected covariates as independent variables. The selected covariates were well-established risk factors for SSI or possible additional factors inflicted due to the nature of the original trial, such as additional interventions from the EPO_2_CH bundle: age, American Society of Anesthesiologists (ASA) physical status score, body mass index (BMI), diabetes, chronic obstructive pulmonary disease, estimated blood loss, GDFT, hyperoxygenation, indication for surgery (benign vs malignancy), the level of wound contamination according to the CDC criteria [16], normoglycaemia, normothermia, procedure duration, sex, smoking, and type of hospital (academic vs non-academic). Based on the estimated PSs, patients who received aqueous chlorhexidine gluconate were weighted as 1/PS and those who received povidone iodine as 1/(1-PS). The balance statistics were checked across all imputation sets by using standardised differences. Appropriate balance was indicated at a standardised difference of less than 0.10 [19]. The E-value was calculated to assess the potential effect of unmeasured confounders [20].

The effect of pIOWI type on SSI was estimated using an unadjusted univariable regression analysis and a weighted univariable logistic regression with a robust variance estimator, applied to each of the 10 imputed datasets. Rubin's rule was used to pool the resulting estimates [21]. The results were presented as risk differences (RDs) and the number needed to treat (NNT), with corresponding 95% confidence intervals (CIs). Odds ratios (ORs) with 95% CI were additionally reported in the supplementary materials. For positive outcomes, the NNT was expressed as the NNT for an additional beneficial outcome (NNTB), while for negative outcomes it was represented as the NNT for an additional harmful outcome (NNTH). The NNTB and NNTH with corresponding 95% CIs were calculated by taking the inverse of the RD. The NNTB can range from 1, representing the maximum possible beneficial treatment effect, to infinity (∞), indicating no treatment effect. The NNTH can range from ∞, indicating no treatment effect, to 1, indicating the maximum possible harmful treatment effect. All analyses were performed with R (version 4.4.0; R Foundation for Statistical Computing, Vienna, Austria), using the packages ‘mice’, ‘MatchThem’, ‘cobalt’, ‘Evalue’.

## Results

From the 699 patients in the EPO_2_CH trial intervention group who received pIOWI with either aqueous chlorhexidine gluconate or aqueous povidone iodine solutions, 254 patients (36.3%) received pIOWI with 1% aqueous chlorhexidine gluconate, while 455 patients (65.1%) received 0.35%–10% aqueous povidone iodine. Baseline patient and procedural characteristics are presented in Table I.

Among the 699 patients, 48 non-organs space SSIs were diagnosed, corresponding to an overall incidence of 6.9%; of these, 39 were superficial and 9 deep incisional SSIs. The crude SSI rate was 8.3% in the aqueous chlorhexidine gluconate group (21 of 254 patients) compared with 6.1% crude rate in the aqueous povidone iodine group (27 of 445 patients).

### Data analysis

**Figure 1:**
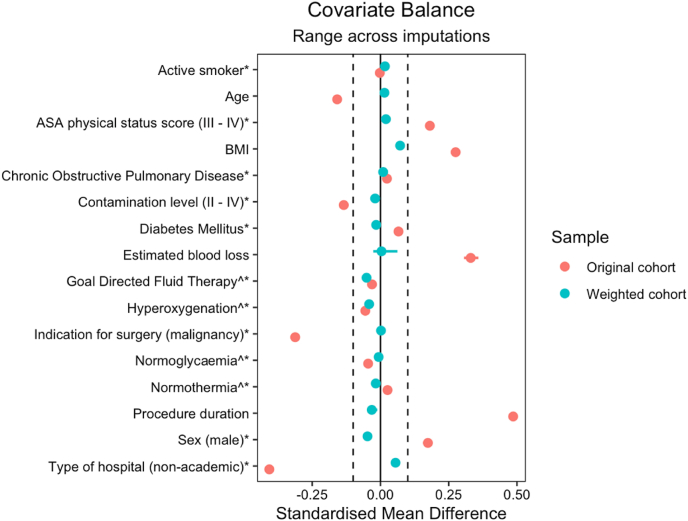
Love plot Standardised mean differences with their range across 10 multiple imputed datasets before (‘Original’) and after inverse probability weighting (‘Weighted’). ∗ Categorised variables ^ Additional interventions from the EPO_2_CH bundle ASA, American Society of Anesthesiologists; BMI, body mass index; EPO_2_CH, Enhanced PeriOperative Care and Health.

Table II and Figure 1 summarise the balance of covariates across all the imputations before and after IPTW. Table III and Supplementary Table A2 show the results of the analysis. Unadjusted regression analysis showed a statistically non-significant difference in SSI rate for pIOWI with aqueous chlorhexidine gluconate (8.27%) compared with aqueous povidone iodine (6.07%) (2.20%, 95% CI: -1.86 to 6.26), with an NNTH 45.5 (NNTH 16.0 to ∞ to NNTB 53.8). Weighted regression analysis showed a statistically non-significant difference in SSI rate (-3.03%, 95% CI: -7.04 to 0.98) for aqueous chlorhexidine gluconate (4.05%) compared with aqueous povidone iodine (7.08%). The NNTB was 33.0 (NNTH 102.0 to ∞ to NNTB 14.2). The E-value was 2.8, indicating that residual confounding could cause the observed association if an unmeasured covariate exists that has a relative risk of at least 2.8 for both SSI and the use of aqueous chlorhexidine gluconate for pIOWI.

**Table II:** Balance across multiple imputations before and after inverse probability of treatment weighting

Characteristic	Before IPTW			Balance after IPTW		
	Povidone iodine	Chlorhexidine gluconate	Mean SMD (range)	Povidone iodine	Chlorhexidine gluconate	Mean SMD (range)
Age in years, mean (SD)	63.78 (12.77)	61.72 (13.29)	−0.1586 (−0.1586 to −0.1586)	63.82 (12.74)	63.65 (11.59)	−0.0134 (−0.0284 to −0.0073)
Male sex (%)	37.75	55.12	0.1737 (0.1737–0.1737)	40.13	33.13	−0.0700 (−0.0728 to −0.0675)
BMI (kg/m^2^), mean (SD)	26.12 (4.40)	27.43 (5.09)	0.2753 (0.2753–0.2753)	26.28 (4.41)	26.49 (4.61)	0.0414 (0.0340–0.0478)
Current smoker (%)	16.45	16.22	−0.0023 (−0.0049 to 0.0013)	17.48	20.00	0.0252 (0.0167–0.0327)
ASA grade III-IV (%)	18.56	36.65	0.1809 (0.1796–0.1836)	22.30	22.76	0.0046 (0.0026–0.0093)
Non-academic hospital (%)	63.15	22.44	−0.4071 (−0.4071 to −0.4071)	53.29	61.42	0.0812 (0.0700–0.0861)
Contamination level III-IV (%)^a^	97.30	83.86	−0.1345 (−0.1345 to −0.1345)	95.79	94.22	−0.0157 (−0.0174 to −0.0144)
Procedure duration in hours, mean (SD)	2.99 (1.44)	3.79 (1.84)	0.4860 (0.4840–0.4875)	3.04 (1.44)	2.94 (1.55)	−0.0588 (−0.0708 to −0.0375)
Estimated blood loss in ml, mean (SD)	281.34 (481.43)	515.02 (876.38)	0.3305 (0.3060–0.3588)	327.56 (546.30)	314.27 (674.99)	−0.0588 (−0.0708 to −0.0375)
Surgery for malignancy (%)	85.17	53.94	−0.3123 (−0.3123 to −0.3123)	78.11	79.67	0.0156 (0.0110–0.0196)
Diabetes (%)	13.48	20.08	0.0660 (0.0660–0.0660)	14.48	12.08	−0.0240 (−0.0261 to −0.0219)
COPD (%)	6.29	8.66	0.0237 (0.0237–0.0237)	6.24	6.78	0.0055 (0.0034–0.0068)
Goal-directed fluid therapy (%)^b^	88.65	85.56	−0.0310 (−0.0440 to −0.0198)	87.92	81.80	−0.0612 (−0.0807 to −0.0486)
Hyperoxygenation (%)^b^	87.87	82.32	−0.0554 (−0.0727 to −0.0451)	85.57	82.58	−0.0299 (−0.0366 to −0.0247)
Normoglycaemia (%)^b^	46.25	41.73	−0.0451 (−0.0456 to −0.0434)	43.43	41.56	−0.0187 (−0.0241 to −0.0124)
Normothermia (%)^b^	64.97	67.56	0.0259 (0.0159–0.0339)	66.53	64.36	−0.0217 (−0.0317 to −0.0099)

The balance of covariates across all imputations is presented as mean standardised mean differences(SMDs) with their corresponding range before and after inverse probability of treatment weighting (IPTW). Pooled means and standard deviations (SDs) are reported for continuous variables and pooled percentages for categorical variables across all imputations.

IPTW, inverse probability of treatment weighting; SMD, standardised mean difference; SD, standard deviation; BMI, body mass index; ASA, American Society of Anesthesiologists; COPD, chronic obstructive pulmonary disease.

a According to the Center for Disease Control wound classification.

b Additional interventions from the EPO_2_CH bundle.

**Table III:** Unweighted and weighted SSI rates

Characteristic	Unweighted results				Weighted results			
	Povidone iodine (%)	Chlorhexidine gluconate (%)	RD (%, 95% CI)	NNT (95% CI)	Povidone iodine (%)	Chlorhexidine gluconate (%)	RD (%, 95% CI)	NNT (95% CI)
SSI	6.07	8.27	2.20 (−1.86 to 6.26)	NNTH 45.5 (NNTH 16.0 to ∞ to NNTB 53.8)	7.08	4.05	−3.03 (−7.04 to 0.98)	NNTB 33.0 (NNTH 102.0 to ∞ to NNTB 14.2)
Superficial	5.62	5.51	−0.11		6.53	2.88	−3.65	
Deep	0.45	2.76	2.31		0.54	1.17	0.62	

SSI, surgical site infection; RD, risk difference; CI, confidence interval; NNT, number needed to treat; NNTH, number needed to treat for an additional harmful outcome; NNTB, number needed to treat for an additional beneficial outcome.

Weighted SSI rates were 7.1% in the povidone iodine group, of which 6.5% were superficial and 0.5% deep SSIs, vs 4.1% in the chlorhexidine group, of which 2.9% superficial and 1.2% deep incisional SSIs. Complete-case analysis showed similar results (Supplementary Table A3).

## Discussion

A non-significant difference in SSI rates between incisional pIOWI with aqueous chlorhexidine gluconate and aqueous povidone iodine was found in our study. Weighted regression analysis showed a difference in SSI rate of −3.03% (95% CI: -7.04 to 0.98) for aqueous chlorhexidine gluconate (4.05%) compared with aqueous povidone iodine (7.08%), with an NNTB of 33.0 (NNTH 102.0 to ∞ to NNTB 14.2). The CIs are very wide, indicating uncertainty of the effect.

Previous studies have evaluated the efficacy of antiseptics as irrigation solutions, demonstrating their effectiveness [4]. However, clinical data regarding the use of aqueous chlorhexidine gluconate for pIOWI are limited. To our knowledge, this is the first clinical study directly comparing aqueous chlorhexidine gluconate with aqueous povidone iodine for incisional pIOWI.

Povidone iodine concentrations may have varied between 0.35% and 10% within our cohort as the specific dilution was chosen at the surgeon's discretion. This is in line with the range of concentrations used in previous clinical trials where the efficacy of aqueous povidone iodine for pIOWI was found [4]. Unfortunately, no data were collected on the specific concentrations used.

The standard concentration of chlorhexidine gluconate in this study was 1%, reflecting routine practice at the initiating hospital and the availability of commercial aqueous solutions in the Netherlands. As the trial was pragmatic, additional dilution at the surgeon's discretion may have resulted in lower concentrations in practice. Most previous clinical studies have used a 0.05% solution [[22], [23], [24], [25]]. Experimental data suggest that this concentration may not achieve maximal bactericidal activity, whereas 1% does [26]. Regarding cytotoxicity, one study reported minimal cell survival after one minute of exposure to 0.02%, with no further reduction at concentrations up to 2% [27]. This indicates that lowering the concentration from 1% to 0.05% may reduce bactericidal activity without meaningfully decreasing cytotoxicity. Nonetheless, there is a paucity of clinical data on the efficacy of various chlorhexidine gluconate dilutions and their potential impact on different types of wound tissue and healing outcomes. In-vitro studies evaluating the cytotoxicity of povidone iodine have produced conflicting results [28,29].

There are growing concerns about the potential for chlorhexidine gluconate to induce antimicrobial resistance [29]. This includes the possibility of contributing to antibiotic resistance through the horizontal transfer of resistance genes between bacteria [30]. Yet, real-world human-derived data have shown no demonstrable change in the susceptibility of SSI-causing pathogens to chlorhexidine gluconate over time [31].

### Limitations

This study is still limited by the non-randomised nature of the data. There are large observed differences in baseline characteristics between patients who were treated with povidone iodine and chlorhexidine gluconate, including differences in hospital type, procedure type, and ASA score. Chlorhexidine gluconate was more likely to be used in academic centres due to common practices where a large proportion of HPB, general surgery (large abdominal wall reconstructions), and gynaecologic procedures (oncologic resections) were performed. All these variables are proxies for the complexity of patients and procedures associated with a higher risk of SSI. IPTW was used to adjust for differences between groups in the best possible way, but there may be some residual confounding. Yet, in the absence of other comparative data, this remains the best available evidence despite its limitations.

Furthermore, we examined how sensitive the observed protective effect of aqueous chlorhexidine gluconate (RD = 3.03%) was to unmeasured confounding using an E-value-based analysis [20]. If this association were truly causal, an unmeasured factor would need to be associated with both treatment use and the outcome by about 2.8% on the RD scale to fully account for the observed effect. Because this value is close to the estimated effect size, the findings appear only moderately robust to unmeasured confounding. Importantly, applying IPTW reversed the direction of the association, indicating that the unadjusted results were strongly influenced by confounding and sensitive to model choice. Although IPTW reduced measured differences between groups, the large baseline imbalances suggest that residual confounding may remain and could plausibly explain the observed association. In addition, there was no standardisation of, or data collected on, irrigation volume or dwell time, possibly leading to procedural variability and variability in outcome, and this may obscure efficacy differences.

The SSI rate for pIOWI with aqueous chlorhexidine gluconate was lower than that with aqueous povidone iodine. However, the difference was non-significant and CIs were wide, indicating uncertainty of the effect. Further research is needed to assess the hypothesis that pIOWI with chlorhexidine gluconate may reduce SSI. Evaluation of its effects on wound tissue and its potential for resistance development also needs further investigation.

## CRediT authorship contribution statement

**Nathan Bontekoning:** Writing – review & editing, Writing – original draft, Visualisation, Validation, Project administration, Methodology, Investigation, Formal analysis, Data curation, Conceptualisation. **Hannah Groenen:** Writing – review & editing, Writing – original draft, Visualisation, Validation, Project administration, Methodology, Investigation, Formal analysis, Data curation, Conceptualisation. **Gerjon Hannink:** Writing – review & editing, Supervision, Methodology, Conceptualisation. **Markus W. Hollmann:** Writing – review & editing. **Stijn W. de Jonge:** Writing – review & editing, Supervision. **Niels Wolfhagen:** Writing – review & editing, Supervision. **Marja A. Boermeester:** Writing – review & editing, Supervision, Methodology, Conceptualisation. **Quirine JJ. Boldingh:** Writing – review & editing. **Wouter J. Bom:** Writing – review & editing. **Linda M. Posthuma:** Writing – review & editing. **Jochem CG. Scheijmans:** Writing – review & editing, Conceptualisation. **Bart MF. van der Leeuw:** Writing – review & editing. **Joost AB. van der Hoeven:** Writing – review & editing. **Jens-Peter Hering:** Writing – review & editing. **Dirk JA. Sonneveld:** Writing – review & editing. **Otto E. van Geffen:** Writing – review & editing. **Eduard R. Hendriks:** Writing – review & editing. **Ewoud B. Kluyver:** Writing – review & editing. **Ahmet Demirkiran:** Writing – review & editing. **Luc RCW. van Lonkhuijzen:** Writing – review & editing. **Marcel GW. Dijkgraaf:** Writing – review & editing.

## Ethics statement

The Amsterdam UMC Medical Ethics Committee (ref. 2015_121) approved the EPO_2_CH trial on 22^nd^ July 2015. Local review boards at participating centres also approved the trial.

## Data access, responsibility, and analysis

H.G. and N.B. had full access to all the data in the study and take responsibility for the integrity of the data and the accuracy of the data analysis.

## Other disclaimers

An earlier version of this work was presented at a conference [32].

## Funding sources

This was a post hoc analysis of the EPO_2_CH trial. The EPO_2_CH trial was funded by the Netherlands Organisation for Health Research and Development (ZonMW) and co-financed by Innovatiefonds Zorgverzekeraars and Ethicon Inc. The funders of the study had no role in study design, data collection, data analysis, data interpretation, or writing of the manuscript**.**

## Conflicts of interest statement

M.A.B. reported receiving institutional grants from J&J/Ethicon and 3M and being a speaker and/or instructor for J&J/Ethicon, 3M, BD, Gore, Smith & Nephew, TelaBio, Angiodynamics, GDM, Medtronic, and Molnlycke. A.M.E. received a European Wound Management grant. No other disclosures (including the use of AI and AI-assisted technologies) were reported. All other authors report no conflicts of interest.
